# ResNet-50 for 12-Lead Electrocardiogram Automated Diagnosis

**DOI:** 10.1155/2022/7617551

**Published:** 2022-04-28

**Authors:** Nizar Sakli, Haifa Ghabri, Ben Othman Soufiene, Faris. A. Almalki, Hedi Sakli, Obaid Ali, Mustapha Najjari

**Affiliations:** ^1^EITA Consulting, 5 Rue du Chant des Oiseaux, Montesson 78360, France; ^2^MACS Research Laboratory RL16ES22, National Engineering School of Gabes, Gabes University, Gabes 6029, Tunisia; ^3^PRINCE Laboratory Research, ISITcom, Hammam Sousse, University of Sousse, Sousse 4023, Tunisia; ^4^Department of Computer Engineering, College of Computers and Information Technology, Taif University, P.O. Box 11099, Taif 21944, Saudi Arabia; ^5^Ibb University, Department of Computer Science and Information Technology, Ibb, Yemen; ^6^LR18ES34 PEESE, National Engineering School of Gabes, Gabes University, Gabes 6029, Tunisia

## Abstract

Nowadays, the implementation of Artificial Intelligence (AI) in medical diagnosis has attracted major attention within both the academic literature and industrial sector. AI would include deep learning (DL) models, where these models have been achieving a spectacular performance in healthcare applications. According to the World Health Organization (WHO), in 2020 there were around 25.6 million people who died from cardiovascular diseases (CVD). Thus, this paper aims to shad the light on cardiology since it is widely considered as one of the most important in medicine field. The paper develops an efficient DL model for automatic diagnosis of 12-lead electrocardiogram (ECG) signals with 27 classes, including 26 types of CVD and a normal sinus rhythm. The proposed model consists of Residual Neural Network (ResNet-50). An experimental work has been conducted using combined public databases from the USA, China, and Germany as a proof-of-concept. Simulation results of the proposed model have achieved an accuracy of 97.63% and a precision of 89.67%. The achieved results are validated against the actual values in the recent literature.

## 1. Introduction

Nowadays, the medical field requires new techniques and technologies in order to evaluate information objectively. According to data from the World Health Organization (WHO), cardiovascular diseases (CVD) represent the leading cause of death globally, where the CVDs account for more than 30% of global mortality each year, and it is estimated to reach around 130 million people by 2035 [1]. Therefore, researchers are developing new methods for preventing, detecting, and treatment of diseases related to the CVD. There are many types of cardiovascular abnormalities, while this study focuses on 26 anomalies, which will be cited later.

The electrocardiogram (ECG) is a recording of the electrical activity of the human heart, which is deemed as a noninvasiveness and real-time exam. It is still one of the essential pillars of the diagnosis of cardiac problems. In recent years, the methods of analysing CVDs have been strengthened by the introduction of imaging procedures, especially the echocardiogram. However, this does not change the importance and usefulness of ECGs, and the parameters could be extracted from this signal. The number of leads on a typical ECG acquisition equipment divides it into 1-lead, 3-lead, 6-lead, and 12-lead ECG. The 12-lead ECG is the most often utilized kind in clinical practice due to its ability to concurrently capture the potential changes of 12 sets of electrode patches attached to the body in standardized places [2]. When comparing to other types of ECG acquisition equipment, 12-lead ECG provides more information on cardiac activity and is frequently utilized in hospital for diagnosis and treatment. In fact, many essential parameters can be extracted from the ECG signal; for instance, the duration and patterns of the various waves, which are indicative of specific cardiac abnormalities.

Professional doctors frequently make ECG analysis and interpretation [3], which is heavily reliant on training, qualifications, experiences, and expertise; thus it is difficult to extract all information from ECG signals [4, 5]. In practice, manual detection of characteristic waves of the ECG signal and classification of heartbeats are difficult and tedious tasks, especially to analyse long-term recordings as Holter examination or ambulatory cases for continuous monitoring in intensive care and resuscitation wards.

With the progress of physical hardware technologies and algorithm, computer-assisted medical diagnoses (CAMD) have become vital in diagnosing CVDs. CAMD based on ECG signals can give professional suggestions or decide instantly by searching for characteristic patterns. It can help doctors make diagnoses and appears to be required due to the huge number of patients in critical care units where they need continuous monitoring. This is how CAMD looked to use the ECG signal to help in cardiac diagnosis. These systems should be easy to set up, upgradeable, accurate, durable, and dependable. The authors of [6] emphasised the importance of using optimization techniques to enhance efficiency for prediction in healthcare applications.

Over the past decades, many techniques for detecting CVDs have been proposed, where some of them are based on signal processing techniques and classification algorithms like support vector machines (SVMs). Deep neural network-based machine learning (ML) and convolutional neural networks (CNN) methods have lately emerged as efficient tools in large applications such as computer vision and natural language processing. Noticeably, coupling ML and DL with healthcare has brought up massive advantages and researchers are striving to find more innovative solutions.

This work aims to classify 27 classes, with ECG signals containing 26 types of CVDs and normal sinus rhythm. This classification where we used four databases contains 42511 ECG records to train, validate, and evaluate models such as CPSC 2018, CPSC 2018-Extra [7], PTB-XL [8], and Georgia [7]. The used dataset contains ECG 12-leads signals, which is a typical ECG set used in clinical cases and hospitals. It is trained with a model based on Residual Neural Networks-50 (ResNet-50) from CNN methods, which is known as one of the most efficient models in classification.

The rest of this paper is structured as follows.  Section 2 presents an overview of related works in the literature; Section 3 represents background information on the interpretation of an ECG.  Section 4 describes the proposed model and our simulation workflow. The proposed ECG classification model results are discussed in Section 5. Finally, Section 6 presents the conclusion and future works.

## 2. Related Work

DL is a subdivision of ML; ML is a subdivision of AI and AI is enabling the machine to act like a human. ML is a way for achieving AI using algorithms trained on data, while DL is inspired by the structure of the human brain or also known as an artificial neural network. The features in ML are picked out with an expert in the domain, whereas in DL they are detected by the neural network without human intervention. That is why DL needs much higher volume of data to be trained to obtain best performance. AI has been shown in numerous experiments to be capable of automatically identifying anomalies registered by an ECG.

Generally, the databases used in papers about ECG diagnosis are public. The first one is from PhysioNet, Massachusetts Institute of Technology-Beth Israel Hospital (MITBIH) [9] which contained only 49 recordings with 30-minute length of each subject, including five classes, normal (*N*), ventricular ectopic (*V*), supraventricular ectopic (*S*), fusion (*F*), and unknown (*Q*). Enabio et al. [10] used MITBIH as a database for ECG classification [11–16]. The second database largely used is Physiological Signal Challenge 2018 (CPSC) [7] which is a public too. It comprises 687,712 lead ECG recordings including eight arrhythmias IAVB (1st degree AV block), AF (atrial fibrillation), LBBB (left bundle branch block), PAC (premature atrial contraction), RBBB (complete right bundle branch block), and SNR (sinus normal rhyme) [17–19]. The third one is Physikalisch Technische Bundesanstalt (PTB) [20] diagnostic database, which contains 54,912 lead ECG records from 290 individuals [21–23]. Selvalingam et al. [24] used private databases to predict ventricular arrhythmias with a DL model, CNN. In addition, Smith et al. [25] collected their data to interpret ECG arrhythmias.

Some studies instead have used more than one. For example, Li et al. [26] used five databases (FANTASIA, CEBSDB, NSRDB, STDB, and AFD). However, they do not combine the data to categorize ECG; instead, they test their model for each data set separately. Zhang et al. [27] used four databases, Acharya et al. [28] constructed 4 sets from a combination of three databases (MITBIH [9], FANTASIA [29], and BIDMC [30]). The study varied on using balanced and imbalanced ones. Wang et al. [31] used two databases (MIT-BIH [9], CPSC2018 [7]) to classify ECG with a recurrent neural network (RNN) model.  Table 1 lists the different databases used in classifying the ECG signals.

In fact, in their workflows, ML methods consider four fundamental steps:

- Signal preprocessing, which includes resampling, noise removal (e.g., band-pass filters), and signal normalization/standardization.
- Heartbeat segmentation, which entails detecting the R-peak (e.g., QRS complex) using algorithms like Pan and Tompkins algorithm [32], the open-source GQRS software supplied by the PhysioNet community.
- Feature extraction, which entails converting raw signals into features that are most suited to the job at hand (e.g., classification, prediction, and regression.).
- ECG signal analysis using traditional machine learning approaches such as multilayer perceptron (MLP) and decision trees.

Even though traditional ML algorithms with handcrafted features have achieved good results for ECG analysis, deep neural network (DNN) methods with the power of automated features extraction and representation learning have demonstrated human-level performance in analysing biomedical signals [33].

DL approaches, on the other side, need a large quantity of data and many parameters to be learnt. Furthermore, most of the suggested methodologies and workflows for evaluating ECG signals are specific to the task, at hand, and cannot be applied to other biomedical topics. Various studies have classified ECG data using a DL approach. Ribeiro et al. [34] created an end-to-end DNN that is capable of identifying six ECG anomalies with a database of 2,322,513 ECG records. The detection accuracy ranges from 83.3% to 100%. This DL model achieves an overall accuracy of 97.57% for the prediction of CVDs. Ahsanuzzman et al. [35] investigated the classification and prediction of a single arrhythmia class, atrial fibrillation (AFib), using ECG signals. A hybrid long short-time memory (LSTM) and RNN was used for this task. Obeidat et al [36] classified six ECG beats classes using a hybrid DL model that combines CNN and LSTM. The hybrid model achieves accuracy and precision of 98.22% and 98.27%, respectively. Further, [37] stressed on utilizing an optimization method to improve efficiency in healthcare applications.

Adedinsewo et al. [38] constructed a CNN model for classifying arrhythmia type left ventricular systolic dysfunction (LVSD) where the attaining accuracy was 85.9%. Xiong et al. [39] decided to train 8528 ECG records from CPSC data, with ResNet-16 model achieving an accuracy of 82%. Zhang et al. [17] used CPSC2018 database, which contained 6877 ECG recordings to build a 34-layer ResNet 1D model in order to detect 9 distinct arrhythmias in 12-lead ECG signals. This model had a classification accuracy of 96.6% for ECG signals.

It can be said that the number of records used is a bit small to train a model of DL; however, as mentioned above, DL needs a much higher volume of data. In this study, we choose to combine four public databases to confirm the efficacy of the model proposed. In this paper, the proposed model has succeeded to diagnose the majority of 27 classes, including 26 CVDs and normal sinus rhythm, which will assist domain experts in identifying patient records, while other researches used ECG to classify just one or two anomalies [35, 38].

## 3. Background Knowledge

It is critical to comprehend electrical cardiac function, since the heart is a mechanical organ that ensures periodic contraction and relaxation. Cells grouped at the nodal level are responsible for an electrical flow that spreads to nearby heart cells (myocardial). Following that, it recontacts to be able to expel blood from other organs.

### 3.1. ECG Principal

**Figure 1:**
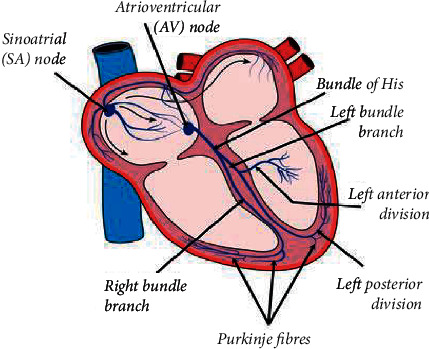
The conductive elements of the heart.

The ECG is a recording of the electrical activity of the heart, which is usually shown as a graph of voltage values vs. time. Electrodes are used to detect electrical changes caused by cardiac muscle cell depolarization and repolarization at a distance from the heart, through the skin. To note, an electrocardiograph is used in this examination.  Figure 1 represents a simplified diagram of the conductive elements of the heart, which consists of conductive tissues which are the bundle of His, Bachmann's bundle, the left and right bundle branches, the Purkinje fibres, and cardiac myocytes themselves. Contractile tissues are the atrial and ventricular wall myocytes. This figure is vital in showing the main components of the heart, so extracting data and signals can be done in more accurate way.

### 3.2. The Foundation of ECG Interpretation

**Figure 2:**
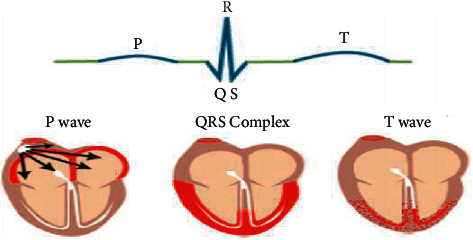
Depolarization/repolarization phases of the heart that are represented electrocardiographically by various P waves, QRS, and T waves.

ECG interpretation includes an assessment of the morphology (appearance) of the waves and intervals on the ECG curve. Therefore, ECG interpretation requires a structured assessment of the waves and intervals.  Figure 2 shows a depolarization/repolarization phase of the heart that are represented electrocardiographically by various P waves, QRS, and T waves.

(i) P wave: This is a result of atrial depolarization, which is initiated by the sinus node. Pacemaker cells at this node carry the signal to the right and left atria. The ECG demonstrates abnormal atrial repolarization.
(ii) QRS complex: This is the average of the inner (endocardial) and outer (epicardial) cardiomyocyte depolarization waves. A typical QRS pattern is formed when endocardial cardiomyocytes depolarize somewhat earlier than the outer layers.
  - The Q wave is the first negative deflection following the P wave. The Q is missing if the first deflection is not negative.
  - The R wave is the positive deflection.
  - The S wave is the negative deflection that occurs following the R wave.
(iii) T wave: It indicates the ventricular repolarization. During the T wave, there is no action in the heart muscle.

Pathologies or abnormalities in ECG analysis are discovered and categorized based on their departure from normal cardiac rhythm. Normal sinus rhythm (NSR) refers to normal cardiac activity in which there is no deviation or change in the morphology of the ECG signal.

**Figure 3:**
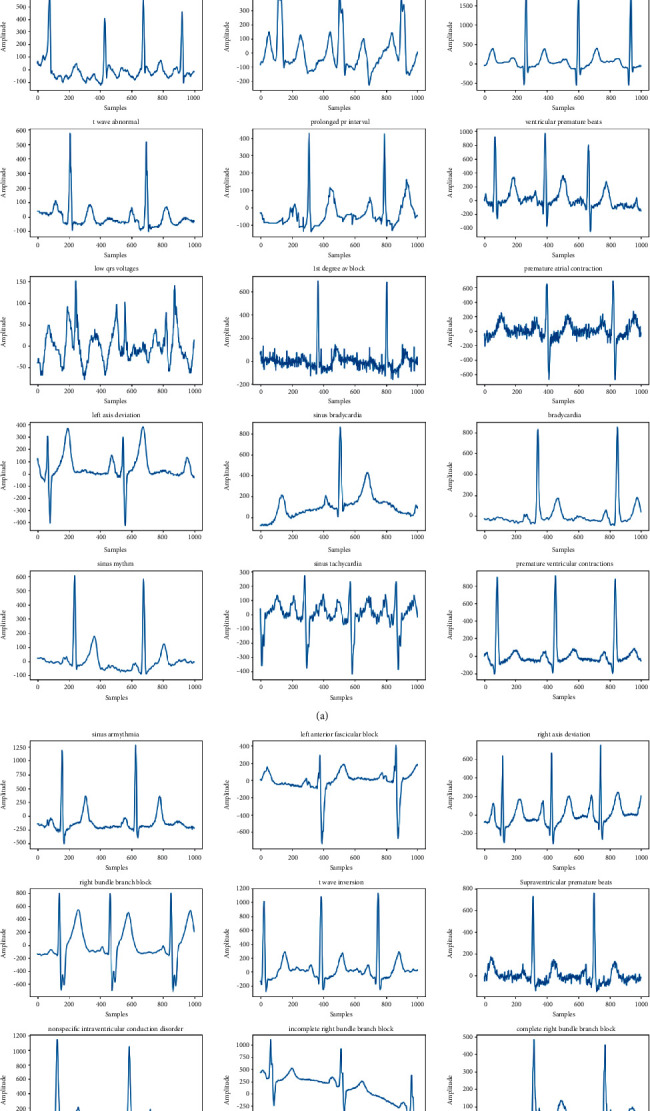
Samples of each class of ECG.

This paper focuses on classifying 27 classes of ECG signal; the classes are 1st Degree AV Block (IAVB), Low QRS Voltages (LQRSV), Right Axis Deviation (RAD), Atrial Fibrillation (AF), Nonspecific Intraventricular Conduction (NSIVCB), Atrial Flutter (AFL), Bradycardia (Brady), Complete Right Bundle, Branch Block (CRBBB), Incomplete Right Bundle Branch Block (IRBBB), Left Anterior Fascicular Block (LAnfb), Pacing Rhythm (PR), Right Bundle Branch Block (RBBB), Premature Atrial Contraction (PAC), Premature Ventricular Contractions (PVC), Sinus Arrhythmia (SA), Sinus Bradycardia (SB), Sinus Rhythm (SNR), Sinus Tachycardia (Stach), Supraventricular Premature Beats (SVPB), Left Axis Deviation (LAD), Prolonged Pr Interval (LPR), Prolonged Qt Interval (LQT), T Wave Abnormal (Tab), T Wave Inversion (Tinv), Left Bundle Branch Block (LBBB), Qwave Abnormal (Qab), and Ventricular Premature Beats (VPB).  Figure 3 shows samples from each of the 27 ECG signal classes.

## 4. Proposed Model

This paper proposes a ResNet model with four databases to classify ECG signals. This section starts by presenting the architecture of model proposed and then highlighting our working method.

### 4.1. Proposed Model Architecture

**Figure 4:**
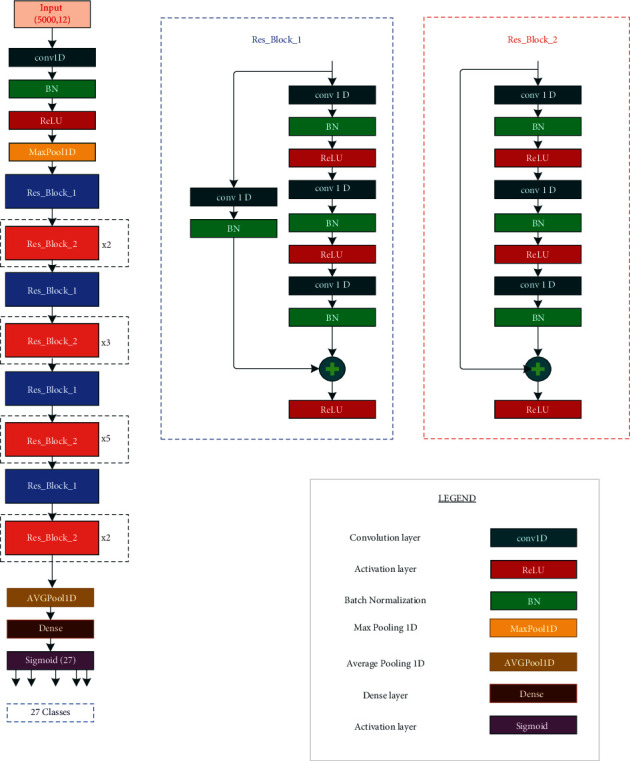
Presentation of the proposed model.

In this paper, ResNet-50 is the proposed model for features extraction. In fact, it combines convolutional neural network for ECG diagnoses.  Figure 4 illustrates an overview of the model architecture. Making the model training tractable has been assured by the residual blocks with shortcut connections. As input, the model takes an ECG signal *x* ∈ ℝ^nsamples×12^. As outputs, the result of the multilabel classification is *ỹ* ∈ ℝ^1×27^.

A 1D convolution layer (conv1D) was applied to these inputs, a batch normalization layer (BN), a rectified linear unit activation layer (ReLU), and a Max Pooling layer. Also, 16 residual blocks have been used to extract deep features. There are two types of residual blocks as follows:

- Res_Block_1 is composed of three Conv1d layers, three BatchNorm1d layers, and two ReLU activation layers. On the one hand, one Conv1d layer and one BatchNorm1d layer are used to match dimensions and skip connections on the other.
- Res_Block_2 is composed only of three Conv1d layers, three BatchNorm1d layers, and two ReLU activation layers.

The Conv1d layers are used for extracting features and the BatchNorm1d layers are used to make the model faster and stable. The ReLU layers are introduced to perform nonlinear activation. The features extracted by the residual blocks are pooled using Average Pooling, where the pooling results are collected and sent to the output layer, which uses the sigmoid activation function to produce predictions.

### 4.2. Dataset Characteristics

The used dataset in this work combines four public databases containing 42,511 recordings of 12-lead ECG. This type of ECG is the most used in clinical cases because of the large amount of information that it generates. These recordings are sampled at a frequency of 500 Hz.  Table 2 describes the characteristics of each database.

**Figure 5:**
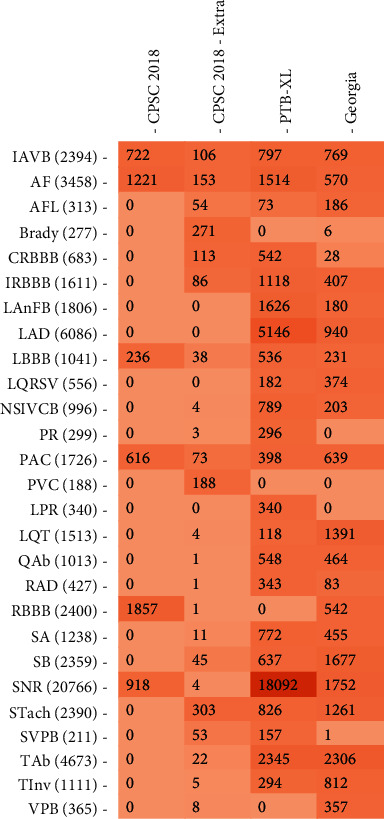
Pathologies distribution in each database.

**Figure 6:**
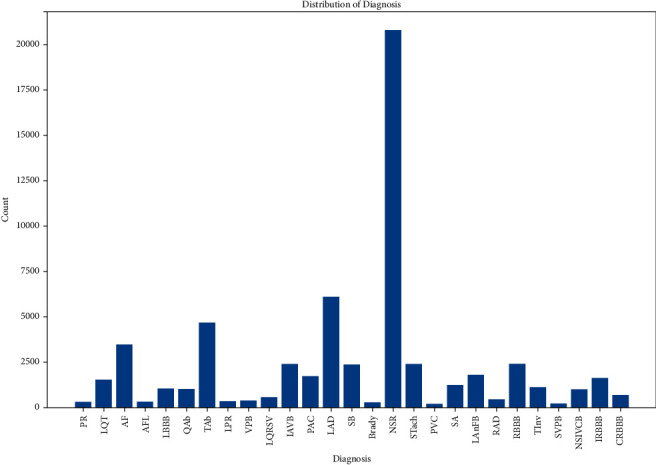
Histogram of pathology distribution in the dataset.

The used dataset in this work contains 27 classes, where 26 classes are of CVDs and a class represents a normal heart state.  Figure 5 shows the distribution of these classes on each database.  Figure 6 illustrates an overview of its distribution in the dataset where a problem of data imbalance and data insufficiency are noticed.

### 4.3. Simulation Workflow

**Figure 7:**
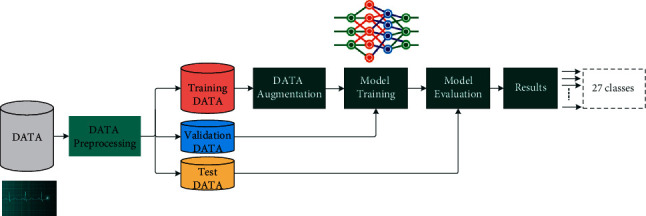
Work methodologies.

Figure 7 illustrates the workflow of the proposed method that has been implemented in our study. Each step of this workflow will be explained in the following subsections.

#### 4.3.1. Data Preprocessing

**Figure 8:**
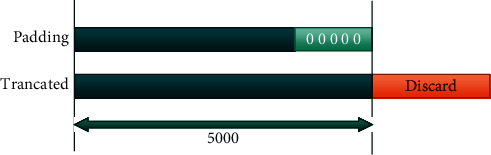
Preprocessing technique.

The length of the signals of the four databases varies from 6 seconds to 60 seconds. Therefore, it has been decided to uniform all the lengths *n* samples. Since the common length is 10 seconds, we set 5000 samples (10 s, 500 Hz as sampling rate). For ECGs recordings having a duration superior to 10 seconds, the first 10 s was kept. Otherwise, signals will be zero-padded until having 10 s as a duration.  Figure 8 describes this preprocessing technique, where in this step, for the signal counting less than 5000 samples will be zero-padded to obtain 5000 samples. For signals containing more than 5000, samples above this value will be discarded.

**Figure 9:**
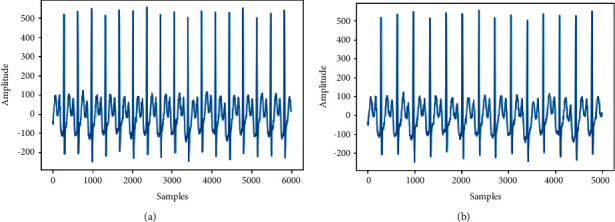
Preprocessing example.

Figure 9 demonstrates in more detail the technique of uniformly reducing the length of an ECG signal, in which we have a signal with a length of 7500 reduced to 5000 to train our model. Data preprocessing is explained as per Algorithm 1.

#### 4.3.2. Data Augmentation

As shown in Figures 4 and 5, the problem of data insufficiency and data imbalance is serious for CVDs. To deal with this issue, amplitude scaling was applied as a data augmentation technique. The creation of realistic data to prevent data scarcity is known as data augmentation. Practically, it enhances the model robustness and lessens the fitting concerns against similar examples [14]. Amplitude scaling is the multiplication of ECG signals by a random factor *α*. This technique aims to compress or stretch the magnitude. The factor *α* is sampled from normal distribution *N* (1, 0.1). The algorithm of amplitude scaling algorithm is shown in Algorithm 2.

#### 4.3.3. Data Split (Train, Validation, Test)

As mentioned in Section 4.2 the dataset used comprises 42,511 ECG records. First, dataset has been split into two sets: test set and training and validation set in the ratio of 0.75 : 0.25. After this, 10-fold stratified cross-validation approach on the training and validation set was applied. This will return 10 stratified folds. These folds will be made by preserving the percentage of samples for each class. This forces the class distribution in each data split to match the distribution in the whole training dataset.

**Figure 10:**
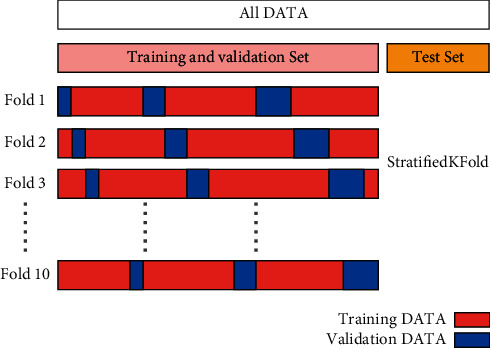
Split data method.

Generally, the training data is dedicated to train the model. The validation data is reserved for optimizing the model. Therefore, a search for the best parametrization without using the test data is done to measure the model performance and allow us to evaluate the model generalization ability. Finally, we obtain a test set and training and validation with 10,627 and 31,884 ECG records, respectively. In addition, the shapes of each training fold and validation fold are 25,507 and 6377 ECG records, respectively.  Figure 10 illustrates an overview of this proposed method.

#### 4.3.4. Training and Evaluation

The trial-and-error approach is used to determine the hyperparameters. In essence, Adam with a learning rate of 10-3 is employed as the optimizer. The binary cross-entropy loss function was used. The optimal values of the hyperparameters of the deep neural network are as follows: the length of the 12-lead ECG input is set to 5000, the batch size is 32, and the number of epochs is equal to 100.

To reduce the learning rate, we used the learning rate scheduler with the following schedule:

(1) $$\begin{array}{c} lr\;=\left\{\begin{array}{cc} \text{lr}, & \text{epoch}<10,\\ \text{lr}.e^{-0.1}, & \text{epoch}\geq 10. \end{array}\right. \end{array}$$

### 4.4. Evaluation Metric

In multiclassification problems, precision and accuracy are commonly used to assess the model's performance. The performance of an algorithm is often measured in terms of four variables for each record. These two performance indictors (accuracy and precision) can be calculated in equations (2) and (3)

(2) $$\begin{array}{c} \text{Accuracy}=\;\frac{\text{TP}+\text{TN}}{\text{TP}+\text{TN}+\text{FP}+\text{FN}}, \end{array}$$

(3) $$\begin{array}{c} \text{Precision}=\;\frac{\text{TN}}{\text{TN}+\text{FP}}, \end{array}$$

where TP denotes True Positive, FP denotes False Positive, TN denotes True Negative, and FN denotes False Negative.

## 5. Results and Discussion

This section presents visual and descriptive discussion based on the proposed model. Additionally, a comparative table has been introduced to compare the proposed work against other studies cited in related works as per Table 3. To note, OVH Cloud has been used with the following characteristic, to train the proposed model.

- Memory: 45 Go
- vCore: 8
- GPU: NVIDIA Tesla V100 16 Go

Precision and accuracy are generally used as two performance indictors to evaluate model performance in multiclassification model. In our situation, precision represents the probability that the model makes the correct prediction, while accuracy is defined as the ratio between the proportion of correct predictions made by the model and the number of total predictions.

In the training and validation phase, the obtained accuracy is 97.63% and 97.58%, respectively. In terms of precision, we obtained 89.67% and 88.85%, respectively. The loss value indicates how well or poorly the proposed model performs after each iteration. For the loss, 3.10^−3^ and 1.27.10^−2^ for each phase were reached as can be seen in Table 4.

**Figure 11:**
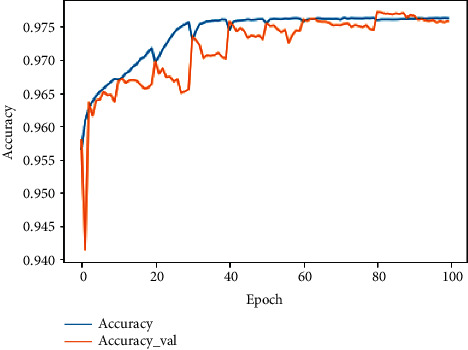
Evolution of training and validation accuracy.

**Figure 12:**
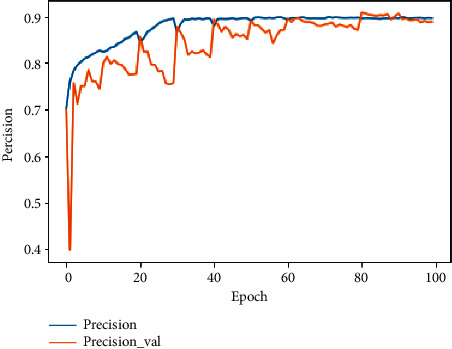
Evolution of training and validation precision.

**Figure 13:**
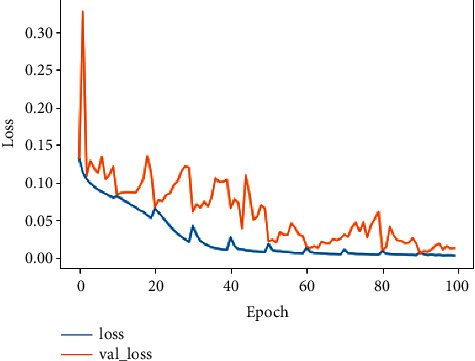
Evolution of the loss in the training and validation.

Because of using stratified 10 folds in the data-splitting step, in the transition from fold to another, the model undergoes a disorder until the stabilization in the last fold. We can observe that, after the 60th iteration, the model progressively converges to reach a stable accuracy, precision, and loss at the 100th iteration. Figures 11–13 demonstrate the evolution of these performance metrics.

It is important for disease diagnosis to improve performance metrics for the correct classification of cardiovascular diseases. ResNet-50 shows better classification performance in comparison to the other studies cited in related works as can be seen in the comparative Table 3.

**Figure 14:**
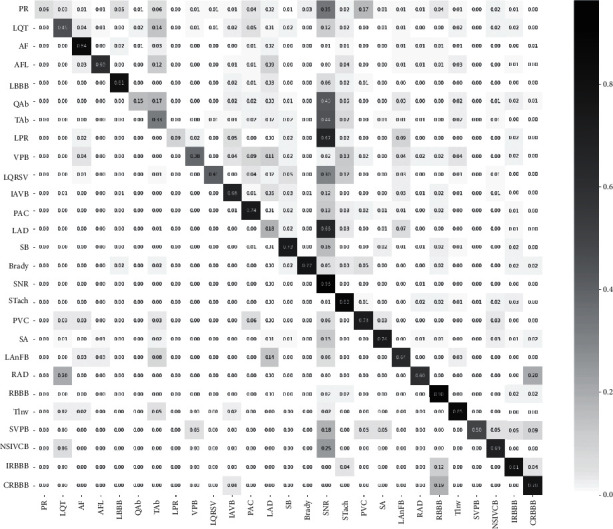
Confusion matrix.

In the evaluation of the proposed model performance, a normalized confusion matrix was created as can be seen in Figure 14, where each row refers to an actual class, while each column represents a predicted class. The proposed model performs well for NSR, RBBB, STach, TInv, AF, IRBBB, and LBBB classes. In effect, their percentage of correct predictions is higher than 80%. It performs moderately for CRBB, Brady, SA, PAC, PVC, and SB classes. Next comes NSIVCB, IAVB, LanFB, AFL, and RAD where the percentage of correct predictions is higher than 60%. For the rest of the classes, like QAb, LAD, and LPR, PR, the model performs badly. This problem of lower predictions is due to the data imbalance even though an amplitude scaling was applied.

Table 5 shows the test results of the proposed model including the incorrect samples (Tests 1 and 2) and correct (Tests 3 and 4) samples that were detected by our model, as well as its prediction and the current state of the ECG.

## 6. Conclusion and Future Work

An effective DL approach based on ResNet-50 has been presented in this paper to classify CVDs. The number of classes that have been considered were 27, where 26 belong to heart anomalies and 1 belongs to normal state. The dataset used in this study combines four datasets collected from three different countries. The achieved results proved the feasibility and the efficiency of the proposed model. The results, also, have been compared and validated against values in the recent published literature. However, the proposed model suffers from high computational complexity and low range of interpretability. Thus, as future research, the proposed approach will be improved to be ideally adapted for wider range of different healthcare applications.

## Figures and Tables

**Algorithm 1:**
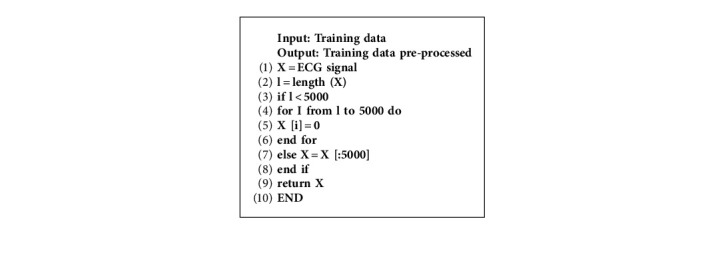
Data preprocessing.

**Algorithm 2:**
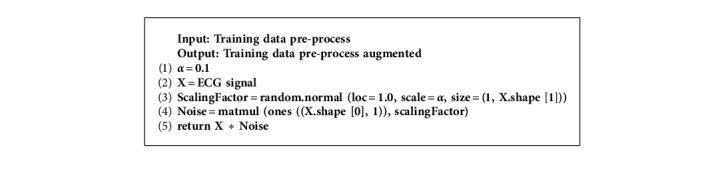
Amplitude scaling.

**Table 1:** Overview of various databases using ECG classification.

Database	Subjects	Records	Duration	Frequency (Hz)	Leads	References
MITBIH [9]	47	48	30 min	360	2	[10–13]
CPSC 2018 [7]	6877	6877	6–60 sec	500	12	[14–16]
PTB [19]	290	549	Not specified	1000	12	[18–20]
Fantasia [29]	40	40	120 min	250	Not specified	[23–25]
BIDMC [30]	Not specified	53	8 min	125	2	[26]

**Table 2:** Description of each database's characteristics.

Database	Sources	Number of ECG recordings	Length of ECG recordings
CPSC 2018 [7]	China Physiological Signal Challenge in 2018	6877 (i) M: 3699 (ii) F: 3178	6 s–60 s
CPSC 2018 EXTRA [7]		3453 (i) M: 1843 (ii) F: 1610	6 s–60 s
PTB-XL [8]	Physikalisch Technische Bundesanstalt	21,837 (i) M: 11,379 (ii) F: 10,458	10 s
Georgia [7]	Georgia	10,344 (i) M: 5551 (ii) F: 4793	10 s

**Table 3:** Results obtained by different research in relation to the proposed work.

Author	Year	Number of records	Model	Preprocessing	Number of classes	Accuracy (%)	Precision (%)
Antonio et al. [34]	2020	2,322,513	DNN	No	6		92.36
Ahsanuzzman et al. [35]	2020	48	LSTM and RNN	Yes	1	97.57	
Obeidat et al [36]	2021	2000	CNN and LSTM	Yes	6	98.22	98.26
Adedinsewo et al. [38]	2020	6613	CNN	No	1	85.9	74
Xiong et al. [39]	2020	8528	ResNet-16	Yes	4	82	
Dongdong et al. [19]	2021	6877	ResNet-34	Yes	9	96.6	
Proposed work	2021	42,511	ResNet-50	Yes	27	97.63	89.67

**Table 4:** Results of the proposed method.

Performance	Results	
	Training phase	Validation phase
Accuracy	97.63%	97.58%
Precision	89.67%	88.85%
Loss	3.10^−3^	1.27.10^−2^

**Table 5:** Test results by the model proposed.

	Test 1	Test 2
Samples	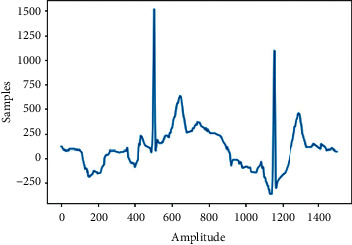	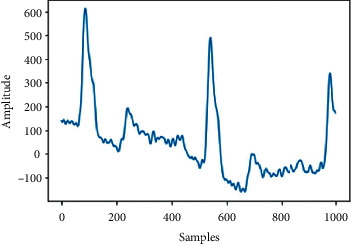
Incorrect	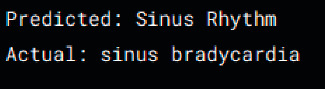	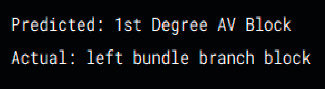

	Test 3	Test 4
Samples	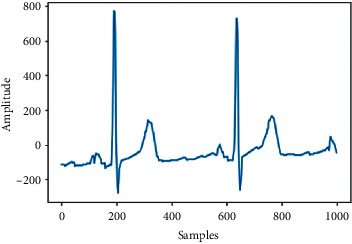	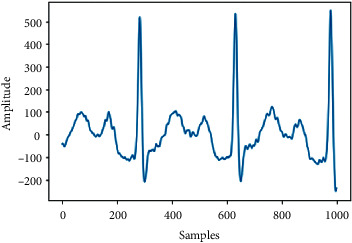
Correct	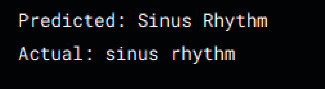	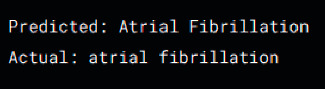

## Data Availability

The data used to support the findings of this study are included within the article.
